# Evaluation of stereoacuity with a digital mobile application

**DOI:** 10.1007/s00417-021-05195-z

**Published:** 2021-04-27

**Authors:** Silvia Bonfanti, Angelo Gargantini, Gabriele Esposito, Alessio Facchin, Marta Maffioletti, Silvio Maffioletti

**Affiliations:** 1 Department of Management, Information and Production Engineering, University of Bergamo, viale Marconi 5, 24044 Dalmine, Italy; 2 Civic Institute of Optometry, Milano, Italy; 3 Institute of Research and Studies in Optics and Optometry, Vinci, Italy; 4 Department of Psychology, University of Milano-Bicocca, Milano, Italy; 5 Humanitas Castelli Clinic, Bergamo, Italy; 6 Degree Course in Optics and Optometry, University of Turin, Turin, Italy

**Keywords:** Binocular vision, Anaglyph colors, Stereo threshold, Stereoacuity, Randot stereotest, Global stereopsis, Digital stereotest

## Abstract

**Purpose:**

Stereopsis is a fundamental skill in human vision and visual actions. There are many ways to test and quantify stereoacuity: traditional paper and new digital applications are both valid ways to test the stereoacuity. The aim of this study is to compare the results obtained using standard tests and the new Stereoacuity Test App developed by the University of Bergamo.

**Methods:**

A group of 497 children (272 males), aged between 6 and 11 years old, were tested using different tests for the quantification of stereopsis at near. These tests were TNO, Weiss EKW, and the new developed Stereoacuity Test App.

**Results:**

A one-way repeated measure ANOVA showed that the three tests give different thresholds of stereoacuity (*p* < 0.0001). Post hoc analyses with Bonferroni correction showed that all tests showed different thresholds (*p* < 0.0001). The lower threshold was obtained by Titmus Stereo Test followed by Stereoacuity App, Weiss MKW, and TNO.

**Conclusion:**

The stereoacuity based on global stereopsis showed that the better values were obtained in order by Stereoacuity Test App, TNO, and Weiss EKW. However, the clinical significance of their values is similar. The new digital test showed a greater compliance by the child, showing itself in tune with the digital characteristics of today’s children.


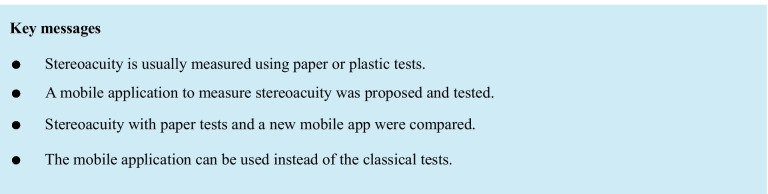


## Introduction

Stereopsis is a fundamental skill in human vision and visual actions and a reduced level of stereopsis has a negative impact on the ability of an individual to perform many tasks [1–4]. Consequently, stereopsis is an important factor in the clinical evaluation of vision [5].

When binocularity is altered for example due to strabismus, anisometropia, or monocular pathology, the stereopsis is disturbed or absent. Moreover, stereopsis together with visual acuity is an important factor in screening children at risk for amblyopia and esotropia [6].

Stereoacuity is defined as the smallest detectable depth difference (threshold) that can be seen in binocular vision [5].

In different studies, stereoacuity has been assessed principally using two methods: anaglyphic and stereoscopic tests that did not require glasses. Anaglyphic tests use polarized or red/green glasses to present two different images at the two eyes, and they are based on printed cards with different symbols or patterns. Some of these tests are the Randot, TNO, and Titmus Fly Test [7].

Conversely, other stereoscopic tests did not require glasses to present two different images at the two eyes. Only few tests are directly stereoscopic like Frisby, and Lang. Both in research and in clinics, all these tests are standard to assess stereopsis. The metrical properties of stereotest as validity and reliability and the usefulness of classical stereotest as a screening are a debated theme [8–10].

However, clinically speaking, especially during screenings, there is a need for a convenient way to measure stereopsis in the clinic. Computers are not comfortable for this use. Some stereotests are available on specific smartphone app stores but their convergence validity (concordance) and agreement with standard tests are not reported. Without these data, the utility of the test is unknown [11].

Another solution is to use a special autostereoscopic smartphone or tablet in which the stereoscopic effect was obtained without the need of glasses but using the autostereoscopic (parallax) technology [12, 13].

Measuring stereopsis with smartphones or in general with digital devices provides several benefits, including a better availability, ease of use, and reproducibility. Moreover, high-resolution displays allow obtaining a small degree of stereopsis. Actual screens with high DPI (dots per inch) are available on market and consequently with these devices a precise evaluation of stereopsis could be performed reaching the threshold of healthy persons.

In this study, we describe and compare a new app SAT (Stereoacuity Test) for Android devices aimed to measure stereoacuity in children. In order to assess the convergent validity of the Stereoacuity Test App, we have decided to compare the results with two other red-green anaglyph stereotests: TNO and Weiss MKW.

## Materials and methods

### Participants

Five hundred forty-six children participated in the study and they were recruited from different school screening programs. Only participants with permission from their parents to take part in the study were enrolled. They are from 6 to 10 years old, 177 6-year-old students, 62 7-year-old students, 74 8-year-old students, 64 9-year-old students, and 98 10-year-old students. Exclusion criteria were the total absence of stereopsis at Lang stereotest I and the absence of one of the tests explained in the “Stimuli and devices” section, used for comparisons. For this reason, forty-nine children have been excluded. The final experimental group was composed of 497 participants (272 males and 225 females), mean age 7.5 years, SD = 1.56, range 5–10.

### Stimuli and devices

For comparison, three stereotests were used. Below there is an outline of each test together with LANG I. LANG I stereotest was used only for the evaluation of the presence/absence of stereopsis. The three stereotests used for the experimental evaluation were all based on red/green dissociation and tested global stereopsis.

#### LANG I

Lang I is an autostereoscopic random dot test. It is a fast test for the screening for the presence of stereoscopic vision in children. The test shows three objects: a star, a cat, and a car. This test was not performed to measure the stereoacuity, but it is only executed to check if the child presents or not stereopsis.

#### TNO

The TNO test (Lameris Ootech BV, Nieuwegein, Netherlands, 13th edition) is a random dot test based on red/green glasses separation to assess global stereopsis. It is composed of 7 boards. The first three boards were used to understand if the stereopsis is present or not, while the other boards measure the stereoscopic sensitivity. Stimulus is a random dot anaglyphic circle in which one sector is lost. They were described as a cake without a slice to the child. The direction of the sector is the answer key, and it could be in one of the four directions: up, down, left, or right. The sensitivity is measured in arcsec and the range of values of stereopsis range from 240 to 15 arcsec.

#### Weiss MKW

The Weiss MKW test is a random dot near vision stereotest based on red/green glasses dissociation. It displays boxes of increasing difficulties. Each box contains a circle, and the user has to point out which circle looks closer. The measurable values range from 480 to 30 arcsec.

### Stereoacuity Test App

**Fig. 1:**
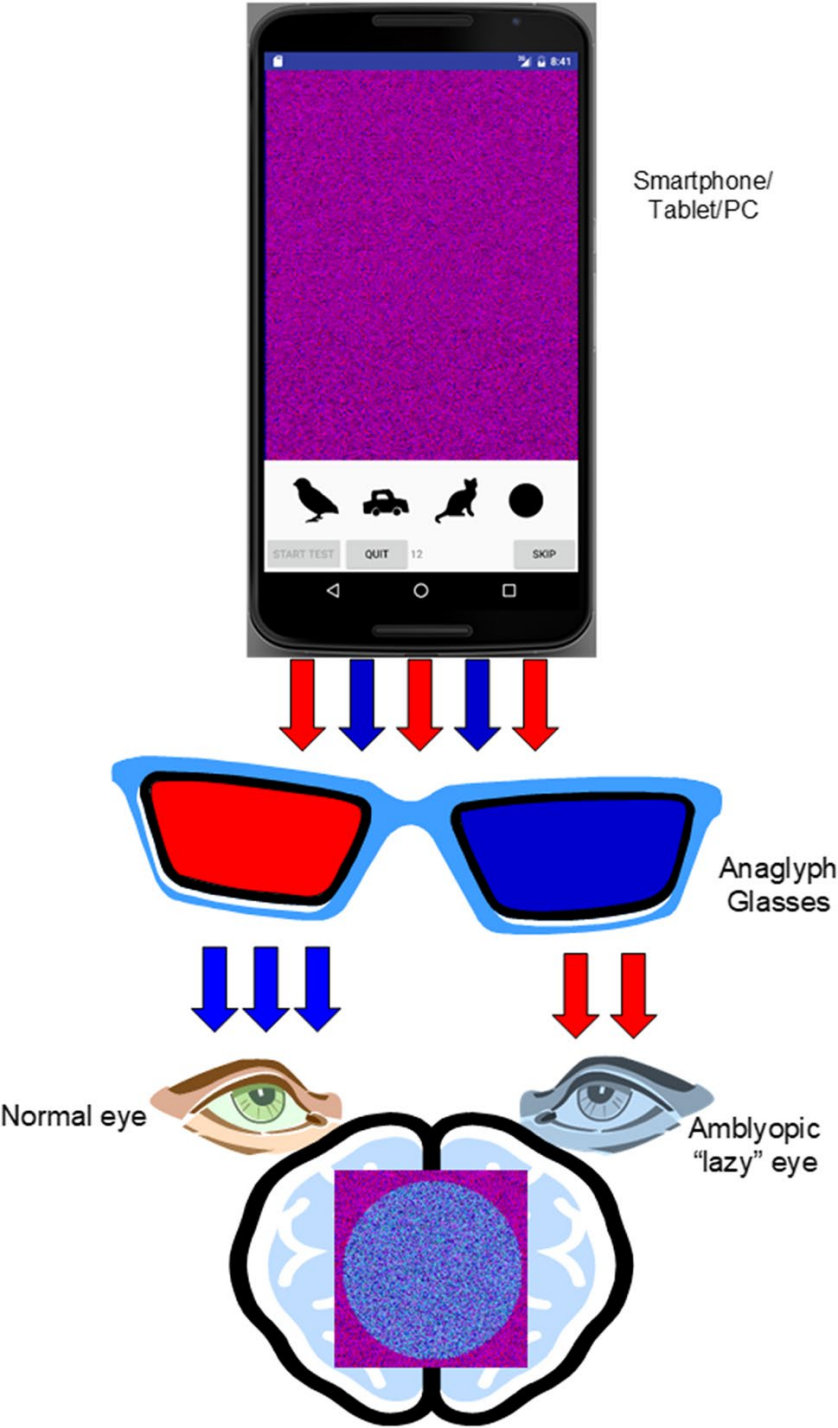
Stereoacuity Test with anaglyph technology

The digital random dot stereotest proposed in this study is an Android application called SAT based on anaglyph technology.^1^ The stereo effect is provided by encoding the images with two different complementary colors. To perform the test, the observer has to wear anaglyph glasses to carry out the image separation. The dots shown on the screen are colored, some of them are visible to both eyes, some dots of the figure to guess are shown to the left eye, and some to the right eye. Usually, the glasses have one red lens and one cyan lens, like those shown in Fig. 1, the red points are seen by the eye with the cyan lens, while the blue points are seen by the eye with the red lens. However, SAT allows the operator to administer the test using different types of anaglyph glasses; the only characteristic required is that the lenses are of two complementary colors.

**Fig. 2:**
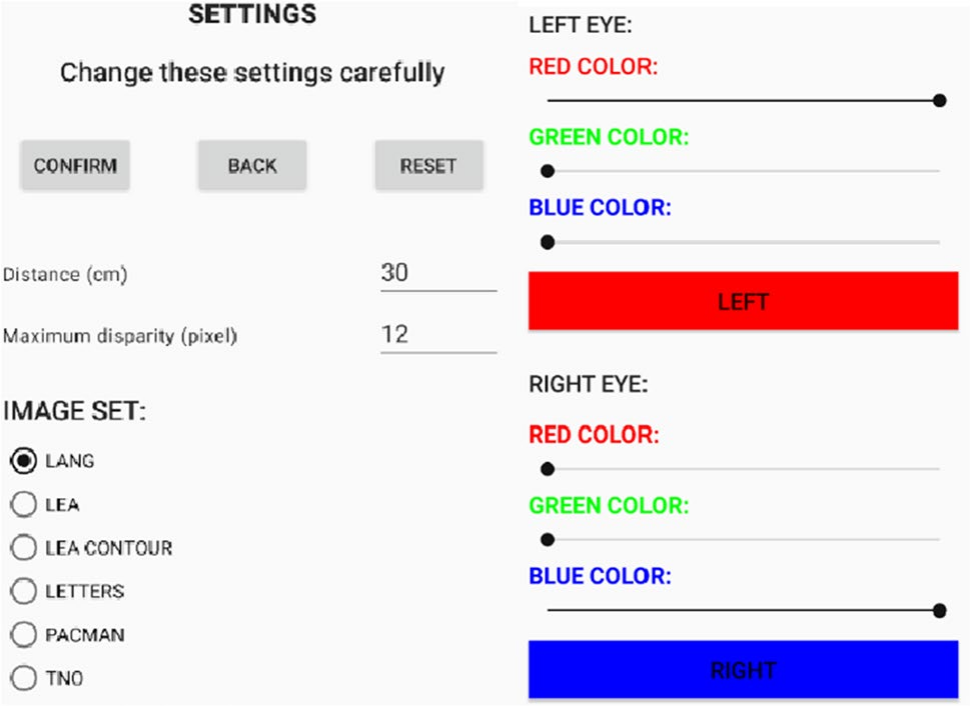
SAT settings

The SAT app allows the selection of the image set (between TNO, LANG, LEA, LEA contours, letters, and pacman), starting disparity, and the distance of the eye from the screen as shown in Fig. 2. Moreover, it allows setting the color of the filters by moving slidebars (see Fig. 2) if necessary.

When the test is started, after a preview of what the shapes will look like, the software randomly chooses a shape in the selected image set and shows the random dot for that shape. The child either says or indicates what it sees and if the guess is right then the software chooses another shape and decreases the angle of disparity. The application selects the next presentation based on a revisited version of Staircase algorithm: the software assesses a certain level of crossed disparity if the child can correctly identify the shapes for that level three times with maximum one error.

Both the minimum measurable stereoacuity angle and all the intermediate stereoacuity levels between the maximum stereoacuity angle till the minimum measurable stereoacuity angle depend on the smartphone resolution (smartphone with higher resolution can measure lower stereoacuity angles compared to smartphone with lower resolution) and on the distance of the child from the screen. The degree of stereoacuity *α* is calculated with a mathematical model explained in [14] by the formula $\alpha ={\mathrm{tan}}^{-1}(d/D)$ where *d* is the disparity between the two anaglyphic images and *D* is the distance from the display.

In order to improve the characteristics of measurement of the test, we have applied the following policies:

1. The shape is randomly chosen every time.
2. The examiner that delivers the test has no cue about which shape is currently displayed.
3. The absence of any monocular cues by using the software both without glasses and with glasses but with one occluded eye was checked. In both cases, the identification of the images was not possible.
4. The shape is shown as image: if the child has difficulties to recognize the shape and identify its name, he/she can simply point his/her finger on the screen.
5. The test has initial learning phase in which no measurement is taken, and the images are shown colored: by this way, the child understands what the shape will look like when the actual measurement is started and the color disappears.
6. When a child fails the test, the test can be repeated with a different set of images. Since images are randomly chosen, the test can be repeated without learning effect (differently from the classical paper tests).

The whole procedure of administration of SAT lasts about 45–60 s, depending on the staircase algorithm and on the response time of children. In this study, SAT was performed using a Huawei p10 smartphone. The screen resolution was 1080 × 1920, and the density was consequently 432 dpi. At 40 cm, the corresponding max resolution of stereopsis presented was 30 arcsec.

### Procedures

Data were collected during different vision screening programs. During these screening programs, other visual parameters (power of glasses used if the child wears glasses normally, monocular and binocular visual acuity, objective refraction, proximal point of convergence) and visual signs (external observation and cover test) were collected. These screenings were performed with different aims, and a standard set of test and instrumentation were not maintained; consequently, other evaluations were impossible to perform. Lastly, the different four stereopsis tests were collected.

The first test administered was the Lang I test; if the result was positive, the child continued with the other tests; otherwise, the stereoacuity measurement was interrupted. This strategy has been applied because the goal of this study was to test the application only on those children who present stereoacuity.

Testing was performed in a quiet room with a uniform illumination of about 350–400 lx. Every child was accompanied by a teacher or a caregiver, and they gave the basic demographic data. Subsequently, the different tests were performed by one or more examiners in a random order. When the child ends the examination, the same teacher/caregiver accompanies the child to the classroom.

Stereotests, SAT included, were positioned on a table 30 cm away with a reading lectern with a fixed tilt of 20 degrees compared to the desk surface without reflection of room lights.

### Statistical analysis

Since participants who did not perceive stereopsis at Lang I test were excluded, the comparisons were performed between TNO, Weiss, and SAT. Stereopsis data were not distributed normally; consequently, all values were transformed with logarithmic function [15].

Firstly, the mean results of stereoacuity were compared (also called bias between tests). A repeated measure ANOVA was performed with the factor test with three levels (TNO, Weiss, SAT). Post hoc comparisons were performed with Bonferroni correction using the “pairwise.t.test” R function with the required arguments (paired and Bonferroni).

Secondly, the agreement between tests with the Bland–Altman procedure was calculated and graphs were reported. Thirdly, the correlation between tests was performed using the intraclass correlation coefficient and specifically the ICC(C,1) [16].

Statistical analyses and graphical representations were performed with R statistical environment [17].

## Results

**Fig. 3:**
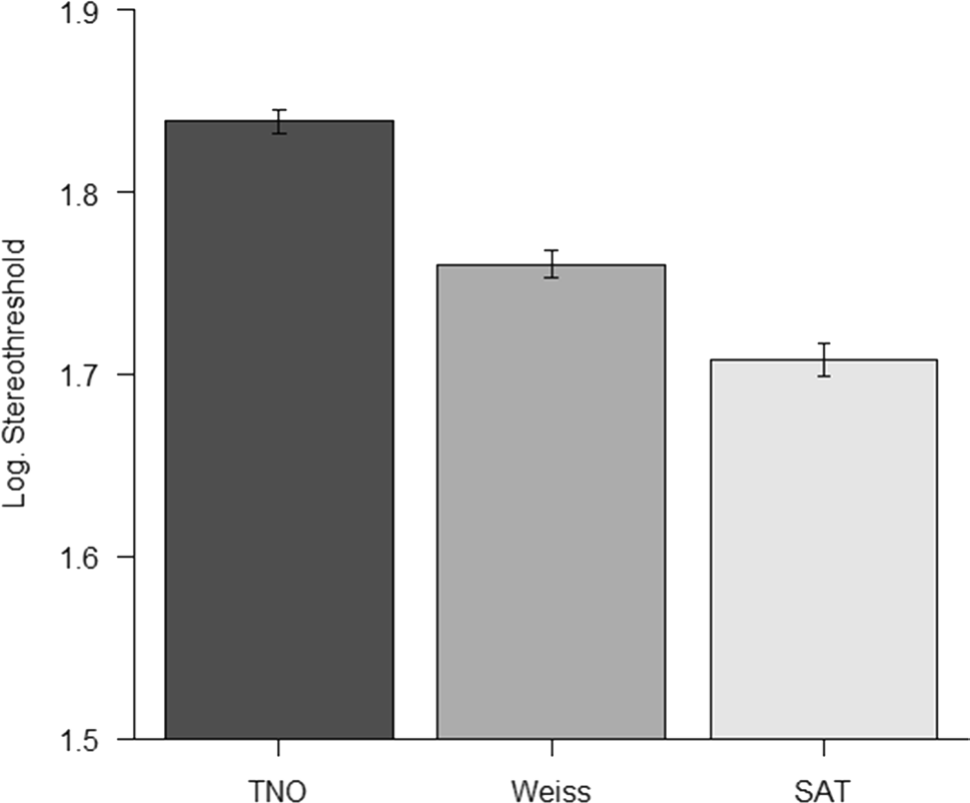
Mean results of the three stereopsis tests in the group of children examined. Thresholds are represented in the log10 arcsec scale

Results of the test comparison showed significant differences between tests F(2,992) = 143.14 *p* < 0.0001 *η*^2^_*p*_ = 0.22. Post hoc comparisons show significant differences between tests (all *p* < 0.0001). Data are displayed in Fig. 3. Retrotransformed in arcsec, the three tests showed a mean value of stereoacuity of 69, 57.6, and 51.1 arcsec for TNO, Weiss, and SAT, respectively.

**Fig. 4:**
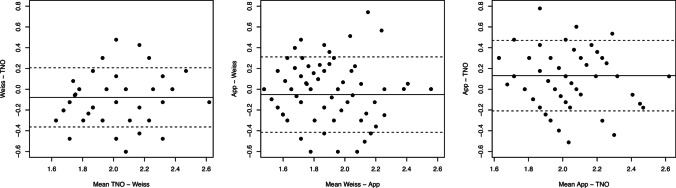
Bland–Altman plot that shows the agreement between tests

In order to show the agreement between tests, three Bland–Altman plots were traced, and limits of agreement (LoA) calculated. Results show that the LoA between TNO and Weiss was 0.28, between Weiss and App was 0.36, and between App and TNO was 0.34. Data are plotted in Fig. 4.

Another form of comparison between tests could be performed taking into consideration the correlation between results [18]. ICC correlation between tests shows a moderate correlation ICC = 0.532 (0.48–0.58), *p* < 0.0001. Single correlation between tests showed even moderate correlations: between TNO and Weiss ICC = 0.58 *p* < 0.0001; between Weiss and SAT 0.49 *p* < 0.0001; and between SAT and TNO ICC = 0.53 *p* < 0.0001.

## Discussion

The aim of this study is to describe and compare the new app SAT with two standard “paper” red/green anaglyph tests like TNO and Weiss stereotests.

First of all, the new digital test has shown a good compliance by the child, despite the longer execution time (around 45/60 s), showing itself in tune with the digital characteristics of today’s children [19]. All children were capable of performing the test and no dropout of testing was found.

Results show that the three tests examined show slightly different results. These values show a statistically significant difference from each other, but clinically irrelevant. The staircase procedure of SAT, together with the response writing in a more precise step than the other two stereotests, has permitted us to obtain higher thresholds. Clinically speaking, all these instruments are permitted to obtain the stereoacuity threshold, with some little difference between each instrument.

It is important to note that all tests measure global stereopsis with random noise stimuli, which is substantially different from measuring local stereopsis with different stimuli like Titmus Stereo Test rings. The local stereopsis, since it presents monocular indices and cues, cannot be compared directly with global stereopsis, and clinically it was not useful to detect microstrabismus [20].

However, the clinical significance of the obtained value is similar, and a common threshold could be considered for all tests. Conversely, since different stereopsis tests have revealed different thresholds, slight difference in reference values and specific normative data will be required [21].

Another advantage of SAT application was the free availability and the easy use in a clinical setting. In fact, the application runs in the large part of recent smartphones with high-resolution display and requires simple anaglyphic glasses. Other techniques, like autostereoscopic method of presentation, require special devices [12, 13].

As previously mentioned, SAT was performed only on children that passed Lang I. As future work, we plan to test the application on patients that do not pass Lang I (due to amblyopia and/or strabismus) to evaluate sensitivity and specificity of SAT. Furthermore, we will test the repeatability of the test performed using the digital application together with the comparison with stereotests that did not require glasses for dissociation like Frisby test and autostereoscopic smartphones.

## Footnotes

1 Available on Google Play Store at https://play.google.com/store/apps/details?id=p3d4amb.sat.app
